# Timely initiation of first antenatal care visit and associated factors among pregnant women in the Amhara Region, North-west Ethiopia: a comparative cross-sectional study

**DOI:** 10.11604/pamj.2023.44.51.33997

**Published:** 2023-01-25

**Authors:** Workaferu Fetene, Alemtsehay Mekonnen, Zemenu Shiferaw, Almaw Genet, Semehal Haile

**Affiliations:** 1 Dangla District Health Department, Awi Zonal Administration, Amhara Region, Dangla, Ethiopia,; 2 Department of Reproductive Health and Population Studies, School of Public Health, College of Medicine and Health Science, Bahirdar University, Bahirdar, Ethiopia,; 3 Department of Midwifery, College of Medical and Health Sciences, Jigjiga University, Jigjiga, Ethiopia

**Keywords:** First ANC visit, associated factors, pregnant women, transformed districts, Amhara Region, Ethiopia

## Abstract

**Introduction:**

timely initiation of the first antenatal care visit is still a major public health problem in Ethiopia, especially in Amhara Region. This study assessed the prevalence of timely initiation of first ANC visit and its associated factors among pregnant women in transformed and non-transformed districts, Awi zone, Amhara Region, North-west Ethiopia.

**Methods:**

a community-based comparative cross-sectional study was conducted from November-December 2020, among 748 women. A multistage-stratified random sampling technique was used. Data were collected by a structured questionnaire. Data analysis was done by SPSS-version 20. Binary logistics regression analysis was used to identify factors. The odds ratios were computed and a p-value < 0.05 was used to declare statistical significance.

**Results:**

the overall prevalence of timely first ANC visits was 40% (95%CI: 36.6-43.7%). It was higher for the transformed districts 46.9% (95%CI: 42.1%-52.5%) compared to non-transformed districts 32.9% (95%CI: 28.3%-37.9%). Higher wealth (AOR=2.17, 95%CI: 1.44-3.27), previous service satisfaction (AOR=1.78, 95%CI: 1.26- 2.51), nearer to the health facility (AOR=3.09, 95%CI: 1.69-5.63), primary education and above (AOR=5.18, 95%CI: 2.99-8.96), knowledgeable mothers (AOR=2.30, 95%CI: 1.38-3.85) and waiting time (< 1-hour) (AOR=1.45, 95%CI: 1.02-2.08) were significantly associated factors.

**Conclusion:**

the prevalence of timely initiation was higher for transformed districts but it is below the WHO target. Higher wealth, previous service satisfaction, being nearer to the health facility, maternal education, being knowledgeable, and waiting time were significantly associated with timely initiation of the first ANC visit. Hence, the district´s transformation should be enhanced. Maternal knowledge, access, and quality of maternal health services should be enhanced.

## Introduction

Antenatal care (ANC) is defined as the routine care of pregnant women provided for the mother during pregnancy to improve the health of the mother and unborn baby [1,2]. ANC is more effective in preventing adverse pregnancy outcomes when sought early in the pregnancy and continued through to delivery [3]. Globally, more than 500,000 mothers die each year from pregnancy-related conditions, of these, ninety-nine percent (99%) of maternal and newborn mortality occur in developing countries [4-6]. The burden of the problem is higher in African countries especially the problem is severe in sub-Saharan African countries, accounting for 50% of all maternal deaths worldwide [4,7,8]. Of the 21 countries with the highest maternal mortality 15 are in sub-Saharan Africa, including Ethiopia. Despite the sustained effort that excelled to decrease the problem, timely ANC visit is still a significant public health problem in Ethiopia, with the low prevalence in Amhara Region [1,9]. Several factors contribute to the timely first ANC visit initiation. Evidence identified that age of mothers, parity, planned pregnancy, maternal education, average household income, media access, knowledge about the time of ANC booking, educational status of the husband, occupation of the husband, the age difference between the mother and the husband, distance from the health facility, family size, marital status, place of residence, parity, abortion, decision-making ability, women´s wrongly perceived ANC initiation schedule, unplanned pregnancy and being free of pain during pregnancy [1,10-13].

Now a time WHO has also adopted a new ANC model that recommends increasing the number of contact from four visits to eight contacts in order to reduce prenatal mortality and to improve women experiences of care, however, the new Antenatal care model still is not practicable in Ethiopia [14,15]. Federal Democratic Republic of Ethiopia Ministry of Health includes the early initiation of antenatal care service in growth and transformation plan- II (GTP-II) by district transformation agenda [16]. Even though there is evidence about the prevalence of early initiation of ANC visits among pregnant women and associated factors in different parts of the country, there is still a gap or not well-known on early initiation of antenatal care visits among pregnant women in transformed and non-transformed districts. In addition to this, the zonal administration is still straggling to control maternal morbidity and mortality by implementing early initiation of ANC visits [17,18]. Therefore, the aim of this study was to assess the prevalence of timely initiation of first ANC visit and to identify the associated factors of timely initiation of first ANC visit among pregnant women, in the transformed and non-transformed districts at Awi zonal administration, Amhara Region, North-west Ethiopia.

## Methods

**Study design, setting and period:** a comparative cross-sectional study was conducted in Awi zonal administration, Amhara Region, North-west Ethiopia; from November 20 to December 20, 2020. Awi zonal administrative zone had nine rural districts and three town administration districts. Of these, one rural district and town administration is categorized as transformed district by the regional authorities.

**Population, sample size determination, and sampling procedure:** all pregnant women who reside in Awi zonal administration were the source population. All pregnant women who were residing in Awi zonal administration at randomly selected transformed and non-transformed districts were the study population.

**Inclusion criteria:** all pregnant women who have a history of ANC and were pregnant in the study period were included in the study.

**Exclusion criteria:** pregnant women those seriously ill during the data collection period were excluded from the study. The sample size was determined by using the double population proportion formula. The following assumptions were considered: 95% confidence interval, 80% power, two comparison population ratio 1: 1, the prevalence of timely initiation of first ANC visits in transformed district (p1=61.8%), the prevalence of timely initiation of first ANC visits in non-transformed districts (p2= 46.8%), was taken from the previous studies done at Bahir Dar Zuria district among pregnant women´s [1]. The final calculated sample size was 764 women, including a 10% non-response rate. Stratified multistage sampling followed by a simple random sampling technique was used to select districts and pregnant women. Stratification of the districts was done based on their transformed and non-transformed status. Of all kebeles (smaller administrative units) in the district, 50% of them were randomly selected from each district. Proportional allocation to population size was made to determine the required sample size in each selected kebele. Finally, 764 households were selected using simple random sampling, thorough computer generation technique.

**Variables:** the dependent variable was “timely of initiation first ANC” measured as a dichotomous variable. The independent variables include; socio-demographic variables, obstetric related variables, health service-related variables, Knowledge, and decision-making power.

**Data collection:** data were collected using a pretested structured questionnaire and observational checklists. The questionnaire was developed after reviewing different available literature. The questionnaire had five parts: socio-demographic variables, obstetric-related, health-related, knowledge-related, and decision-making power. The questionnaire was developed in English, then translated into the local language, then back to English to check its consistency. To assure the quality of data and avoid biases; a properly designed questionnaire was used, one-day training was given for the data collectors and supervisors, a pre-test was done on 5% of the sample size, and collected questionnaires were checked for completeness on daily basis.

### Measurement

**Timely first ANC visit (early):** pregnancy-related care received from a skilled health care professional less than equal to 16 weeks of gestation [19].

**Transformed District:** high performing or meets the following interrelated criteria namely: model kebele (≥ 80%), high performing primary health care units (PHCUs) (≥ 80%), Community health insurance (CBHI =80%), and district management standard performance (=80%) in the last 6 months.

**Non-transformed district:** the low performance of the four interrelated criteria namely model kebele (< 80%), high performing PHCUs (< 80%), Community health insurance (CBHI < 80%) and Woreda management standard performance (<80%) in the last 6 months [20].

**Kebele:** is the smallest administrative unit in Ethiopia.

**Knowledge:** assessment of respondent´s understanding about timely initiation of first ANC visit, according to listed questions. **Knowledgeable:** if respondent´s response score between 80%-100% from knowledge measuring timely initiation of first ANC visit questions. **Fairly knowledgeable:** if respondent´s response scoring from 50%-79% of knowledge measuring timing of first ANC visit questions. **Less-knowledgeable:** if respondent´s response score < 50% of knowledge measuring questions [16].

### Data procession and analysis

After the data collection, the questionnaires were reviewed and checked for completeness. Then, data were coded and entered in Epi data version 4.6 and exported to SPSS version 20 for analysis. Data were explored for missing values and managed accordingly. Descriptive statistics were used to describe data by using tables and graphs. Categorization was done for continuous variables using information from different kind of literatures and re-categorization was done for categorical variables accordingly. Communality value > 0.5, Kaiser Meyer Olkin (KMO) (sampling adequacy) with P-value > 0.05, and complex structure factor (Eugene value) greater than 1 was considered. Binary logistic regression analysis using Bivariate and multivariable models was done to identify significantly associated variables with the dependent variable. Model fitness was checked using the Hosmer-Lemeshow test with (P-value < 0.05). A p-value < 0.25 was considered to retain variables for multivariable logistic regression model. A backward stepwise logistic regression model was used during multivariable logistic regression to control confounding effects. Crude and Adjusted odds ratios with 95% confidence intervals were calculated for each independent variable to measure the strength of associations between outcome and independent variables. A p-value < 0.05 was considered as the cut-off point for declaring statistical significance.

**Ethical considerations:** a written ethical approval letter was taken from Bahir Dar University Research Ethics Review Committee. Moreover, the support letter was secured from Awi zonal health department. Written consent was taken from each study participant. Each woman was informed about the objective of the study, the confidentiality of their data, and the right to refuse participation. To ensure confidentiality, the name of the interviewee was not written on the questionnaire. Each respondent was assured that the information provided by them as confidential and used only for research. Pregnant women who have any dangerous signs were referred to the nearest health facility.

## Results

### Socio-demographic characteristics of the respondent

A total of 748 pregnant women were surveyed, providing a response rate of 97.9% and the remaining 2.1% were non-responses. An equal proportion of respondents were taken from transformed and non-transformed districts. The mean age of women was 36.49 (SD (standard deviation ±4.15)) and 35.1 (SD±5.14) years old in the Transformed and non-transformed districts, respectively. Almost majority of pregnant mothers 729(97.5%) were orthodox Christians. Similarly, nearly all pregnant mothers 366 (97%) in transformed and 364(98.1%) in non-transformed districts their occupation were farmers. Additionally, all pregnant mothers who were lived in transformed and non-transformed districts were married (Table 1).

**Table 1:** socio-economic and demographic characteristics of about timely first ANC visit among pregnant mothers from transformed and non-transformed Districts, Awi zonal administration, Amhara Region, North-west Ethiopia, 2020 (n=748)

Characteristics	District category code				Total		Chi-square
	Transformed (n=377)		Non-transformed (n=371)				
	Frequency	%	Frequency	%	Frequency	%	P-value
**Age of pregnant mother**							
>= 25 years	21	5.7	31	8.4	52	7	0.00
25-34 years	313	83	263	71	576	77	
>= 35 years	43	11.3	77	20.6	120	16	
**Education status of pregnant mother**							
No formal education	315	83.6	340	92	655	87.6	0.001
Primary and above	62	16.7	31	8	93	12.4	
**Educational level of husbands(n=731)**							
No formal education	306	81	330	89	636	85	0.03
Primary and above	71	19	41	11	112	15	
**Household family size**							
< 5	176	46.7	153	41.2	329	44	0.134
>5	201	53.3	218	58.8	419	56	
**Presence of < 5 children**							
Yes	326	93.7	258	73.3	584	83.4	0.000
No	22	6.3	94	26.7	116	16.6	
**Wealth index**							
Poor	155	41	171	45	326	44	
Medium	106	28	109	29	215	29	0.150
Higher or reach	116	31	91	26	207	27	

### Obstetric, and health service-related factors

In this study, all of the respondents reported that they had a previous history of at least one pregnancy. More than two-thirds of the respondents 319(85%) from transformed districts and 336 (89.6%) from non-transformed districts had one and above parity. Regarding the planning of the current pregnancy, 327 (86%) of respondents from transformed districts and 360(97%) of respondents from non-transformed districts had planned pregnancy. All of the study participants reported that they were attending antenatal care follow-up at governmental health facilities (i.e. health posts, health centers, and primary hospitals). Almost, all transformed and non-transformed districts' health facility maintains pregnant mothers´ privacy, 99.1% and 98.6% of pregnant mothers perceive that health facilities protect their privacy in the transformed and non-transformed districts. Regarding satisfaction level, 248(65.7%) of study participants from transformed and 198(53.4%) of participants from non-transformed districts reported that they were satisfied with the service (Table 2).

**Table 2:** obstetric and health service related factors among pregnant mothers from transformed and non-transformed Districts, Awi zonal administration, Amhara Region, North-west Ethiopia, 2020 (n=748)

Characteristics	District category code				Total		Chi-square
	Transformed(n=377)		Non-transformed(n=371)				
	Frequency	%	Frequency	%	Frequency	%	P-value
**Parity**							
Zero	58	15	26	9	84	11	0.47
One & above	319	85	336	91	641	89	
**History of still birth**							
No	345	83,6	360	85.9	705	85.4	0.001
Yes	32	16.7	11	14.1	43	14.6	
**History of abortion**							
No	351	93	350	94	701	94	0.486
Yes	26	7	22	6	48	6	
**Got advice for going to HF**							
No	44	11.7	66	17.8	104	14	0.134
Yes	333	88.3	305	82.2	638	86	
**Current pregnancy planed**							
No	327	86	360	97	687	92	0.000
Yes	50	14	11	3	61	8	
**Any accompanying person ANC visit**							
No	122	32	146	39	268	36	0.046
Yes	255	68	225	61	480	64	
**Distance from home to HF**							
≤ an hour	345	92	313	84	658	88	0.003
>an hour	32	8	58	16	90	12	
Waiting time							
≤half hour	283	75	146	39.4	429	57.4	0.000
>half hour	94	25	225	60.4	319	42.6	
**Satisfaction level**							
Satisfied	248	65.7	198	53.4	446	59.6	0.001
Not- satisfied	129	34.3	173	46.6	302	40.4	

**Figure 1:**
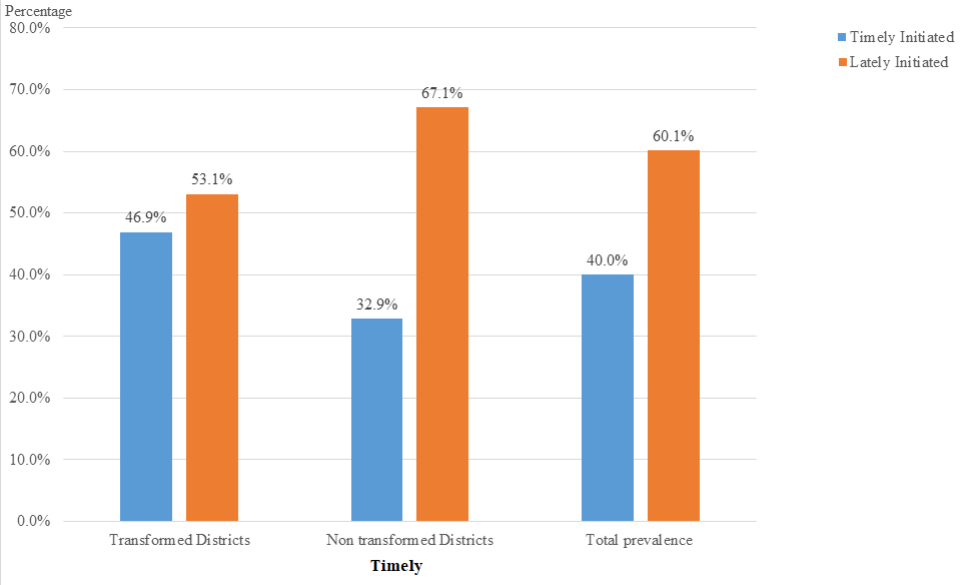
prevalence of timely first ANC visit among pregnant mothers from transformed and non-transformed Districts, in Awi zonal administration, Amhara Region, North-west Ethiopia, 2020

**Prevalence of timely first ANC visit:** seven hundred forty-eight pregnant mothers were interviewed for a timely first antenatal care visit (ANC). The overall prevalence of early initiation of the first ANC visit in the two districts was 40% (95% CI: 36.6, 43.7%). The prevalence found to be 46.9% (95% CI: 42.1%, 52.5%) in the transformed district and 32.9% (95% CI: 28.3%, 37.9%) in the non-transformed district (Figure 1).

### Factors associated with timely first ANC visit

The association of each independent variable with the timely initiation of the first ANC visit was assessed by using binary logistic regression. Initially, bivariate analysis was done and those variables with a P-value below 0.25 were identified as candidates for multivariable analysis. In multivariable logistic regression analysis; wealth, service satisfaction, distance to the health facility, maternal education, waiting times, and mother´s knowledge were significantly associated with the timely imitation of the first ANC visit. Pregnant mothers whose waiting for timing less than or equal to half-hour were 1.459 times likely (AOR = 1.459, 95% CI: 1.021, 2.084) to timely initiate the first ANC visit than their counterparts. Those pregnant mothers who come from households that have higher and medium wealth assets were two times likely (AOR = 2.169, 95% CI: 1.441, 3.265) to timely initiate the first ANC visit than mothers from households with poor wealth assets. The odds of timely initiation of first ANC visit was five times likely 3.093 (AOR = 3.093, 95% CI: 1.697, 5.639) for those pregnant mothers who were taking one hour and below to travel from home to health facility than who those mothers who were taking more than an hour. Pregnant mothers with primary education and above were five times likely (AOR = 5.180, 95% CI: 2.993, 8.965) to timely initiate first ANC visit than those pregnant mothers with no formal education. Knowledgeable pregnant mothers were two times more likely (AOR = 2.305, 95% CI: 1.380, 3.850) to timely initiate first ANC visit than less knowledgeable pregnant mothers (Table 3).

**Table 3:** pooled logistic regression analysis of associated factors of timely first ANC visit among pregnant mothers from transformed and non-transformed Districts, Awi zonal administration, Amhara Region, North-west Ethiopia, 2020 (n=748)

Characteristics		Timing of ANC visit (n=748)			
		**Early**	**Late**	**COR (95%CL)**	**AOR (95%CL)**
**Pregnant mothers living place**	Transformed	177	200	1.806(1.43, 2.429)	0.911(0.609, 1.363)
	Non-transformed	122	249	1	1
**Wealth index of households**	Rich	89	118	1.918(1.331, 2.766)	2.169(1.441, 3.265)***
	Medium	118	97	3.094(2.157, 4.439)	3.504(2.347, 5.230)***
	Poor	92	234	1	1
**Satisfaction level of pregnant mothers**	Satisfied	141	161	1.596(1.185, 2.150)	1.784(1.266, 2.514)**
	Not-satisfied	158	288	1	1
**Decision making power**	Yes	279	402	1.631(0.946, 2.843)	1.854(0.982, 3.497)
	No	20	47	1	1
**History of abortion**	Yes	26	22	1.848(1.027, 3.327)	1.886(0.954, 3.731)
	No	273	427	1	1
**History of still birth**	Yes	24	19	1.975(1.062, 3.674)	
	No	275	430	1	
**Distance from the health facility**	≤ an hour	328	275	1.975(1.207, 3.230)	3.093(1.697, 5.639)**
	> an hour	66	24	1	1
**Husband permission for ANC booking**	Yes	271	393	1.379(0.854, 2.227)	
	No	28	56	1	
**Husband participation on ANC booking**	Yes	90	98	1.542(1.105, 2.153)	1.435(0.981, 2.101)
	No	209	351	1	1
**Current pregnancy**	Planned	33	28	1.865(1.102, 3.158)	
	Unplanned	266	421	1	
**Parity**	Zero	41	43	1.500(0.952, 2.366)	
	One & above	258	206	1	
**Educational level of mothers**	Primary & above	61	32	3.340(2.116, 5.272)	5.180(2.993, 8.965)***
	No-formal education	238	417	1	1
**Knowledge of pregnant mothers**	Knowledgeable	48	43	2.635(1.666, 4.170)	2.305(1.380, 3.850)***
	Fair	118	92	3.028(2.156, 4.253)	3.347(2.296, 4.883)***
	Less	133	314	1	1
**Waiting time**	≤ ½ hour	189	240	1.496(1.109, 2.018)	1.459(1.021, 2.084)*
	>½ hour	110	209	1	1

* = P-value < 0.05, ** = P-value < 0.01, *** = P-value < 0.001

## Discussion

The government of Ethiopia in collaboration with different partners has implemented different types of interventions to alleviate pregnancy-related maternal and child morbidity and mortality. The presence of these interventions improve maternal health by early initiation of ANC visits. According to the findings of the current study, the overall prevalence of timely initiation of the first ANC visit is still lower. The study also tried to compare the prevalence of timely initiation of first ANC visit between the two areas (transformed and non-transformed districts) and differences were seen between them, although it was not statistically significant.

The possible explanation for the absence of statistically significant variation might be due to the similarity of pregnant mothers´ knowledge about the timing of the first ANC visit. Similarly, the pregnant mother´s decision-making power was similar in the transformed woreda and non-transformed districts. Additionally, nearly 1/3^rd^ of the pregnant mothers in both settings had good knowledge about the timing of the first ANC visit. Moreover, the educational level of pregnant mothers in transformed and non-transformed woreda was similar. The other possible explanation for the absence of significant variation between the two areas might be due to the fact that the government implemented a similar initiative to increase the early initiation of the first ANC visit that may have a significant effect on both study areas.

The finding of the study was also compared with other studies done in different parts of the country and abroad. The finding was found to be comparable with the study conducted in central Tigray, Addis Ababa, Boditi town, Cot Devoir, and Mali [9,10,21,22]. However, the finding was lower than the previous study conducted in Bahir Dar Zuria, Burundi, Ghana, South Africa, Niger, Malesia, and Pakistan [22-25]. Such prevalence variations in timely initiation of first ANC booking might be due to lower level current pregnancy planning (=14%) in both areas. If the pregnancy is planned the pregnant mothers were early initiated their first pregnancy which helps to keeps mothers and newborn babies as healthy as possible. As well as, 83.5% and 92% of transformed and non-transformed woreda pregnant mothers were not followed formal education.

The prevalence of timing of first ANC visit was higher than the previous study done on the other parts of the country like Debremarkos (33.4%), Gonder town (35.4%), Arsi zone (32.5%), kembata tembaro zone (31.4%) and 30% in Dilla district [26-30]. In the same way, the prevalence of timing of the first ANC visit was higher than the study done in Kenya, Malawi, Uganda, South-Eastern Tanzania, Ghana, and Zimbabwe [22,25,31-33]. The possible explanation might be due to the improvements in access to health service interventions given in recent years that can improve the early initiation of first ANC visits. On the other way, economic status and the time gap between the studies might bring such differences. The other difference could be due to the comparative nature of this study that includes the transformed woreda which may increase the prevalence of early initiation of first ANC visit in this study.

Pregnant mothers who are lived within short distances from the health facility were more likely early initiate ANC visits than those who travel more than one hour. The finding of the study is consistent with other studies done in Bahir Dar Zuria woreda, Cameroon, and Pakistan [1,34,35]. This might be due to pregnant mothers impose them for extra costs for transportation. In addition to this, it may women living in a developing country spent more time for other activities like drawing water, household chores, rearing children, and for agricultural activities rather than their health. As a result, they didn´t think to attain the health facility for receiving ANC services because they have no time to go there.

This study also shows that pregnant mothers who come from higher-income households had better odds of early initiation of first ANC visit than the poorest. This finding is consistent with Addis Ababa, North West Ethiopia, Kenya, India, and Brazil [31,36-39]. In fact, Ethiopia has declared ANC services are provided for free to all pregnant mothers in order to avoid the financial barrier. However, this is not enough to totally avoid the financial barriers of seeking healthcare during pregnancy. The cost that comes along with traveling to reach distant health institutions could hinder the need and make women reluctant for early initiation and subsequent visits. Mostly they don´t travel alone to a distant health facility, rather either with their husbands or children. Affording for traveling and other related costs may be another barrier even if the services are provided for free.

This study also revealed that a pregnant mother´s educational status was positively associated with the timing of the first ANC visit. The pregnant mothers who had educational status primary and above had more likely early initiate of first ANC visit than those who did not attend formal education. The finding has also become consistent with previous studies done in southern Ethiopia, Soddo town, Kenya, Ghana, and Sindh Pakistan [21,25,26,32,40]. This is might be pregnant mother´s educational status is higher had a better understanding of information about the importance of timely booking of the first ANC visit. As well as it gives a chance to get information about ANC visit time and services delivery to allow or tell their wives to visit health facilities early.

On the other hand, this study also revealed that women who had knowledge on the timing of ANC visits were more likely to early initiate the ANC services than that less knowledgeable ones. The finding was supported by other studies done in Bahir Dar Zuria, Southern Ethiopia, Arsi zone, and Dilla district [12,21,31,29]. It might be pregnant mothers who had knowledge of any risk that was found to be an important factor for their ANC booking. As well as increasing awareness of women regarding the potential health problems that they may encounter during their pregnancy improve their service seeking timing an inadequate understanding of health threats during pregnancy. So, health education related to the potential danger signs of the major causes of mortality in women could have an important input in improving their utilization of care during pregnancy.

Pregnant mothers who had reported that shorter waiting times for getting the services were more likely to start first ANC booking early than those who complain of long waiting times. Similarly, this study also reveals that pregnant mothers who were satisfied with the service were had better odds of early initiation of the first ANC visit than the unsatisfied. This study was in line with the other studies done in different parts of our country Hadya zone and Dila town [41,42]. Studies revealed that the quality of previous service and satisfaction influence the current timing of the first ANC booking [32]. As well as, pregnant mothers complained about waiting time and the service satisfaction it hinders early initiation of the first ANC visit.

The other predictor variable in this study was planning or wanting of the pregnancy. Pregnant mothers who those who are planned the pregnancy were more likely to start the first ANC booking early than not'> pregnant mothers complained about waiting time and the service satisfaction it hinders early initiation of the first ANC visit. The other predictor variable in this study was planning or wanting of the pregnancy. Pregnant mothers who those who are planned the pregnancy were more likely to start the first ANC booking early than not-planed. This is supported by previous studies [1,41,42]. This might be due to when the mother prefers the pregnancy, she is eager to keep the health of the baby. Due to that, they are excited to attain the follow-up earlier.

Pregnant women who participated in at least one household decision-making factor had higher odds of the early initiation of the first ANC visit than counterparts. This study is also in line with the previous studies [26,29,41]. The possible reason for this pregnant mother had a chance to decide on her own health. Because of this pregnant mother early initiates an ANC visit to the health facility. In this study variables like parity, history of abortion, and stillbirth are not statistically associated with the timing of the first ANC visit. But, there were independent predictors in the other studies [32,43,44].

### Strengths and limitations

**Strength:** this study has assessed one of the venerable groups (pregnant mothers) at community level.

**Limitation:** the data was collected by interviewers that can potentially introduce social desirability bias.

## Conclusion

The prevalence of timely initiation of first ANC visit among pregnant mothers was lower than the world health organization (WHO) standard and showed the difference between transformed and non-transformed districts, although it was not statistically significant. This study has found that initiation of first ANC visit was significantly associated with wealth index, the average distance from the health facility, service satisfaction, educational level of pregnant mothers, waiting for time, and knowledge of pregnant mothers. Hence, this study suggests emphasis should be given to the modifiable factors identified by this study; and transformation of the districts should be kept active. Access and quality of maternal health services, and enhancing maternal knowledge on basic maternal health services including ANC should be stressed.

### What is known about this topic

- *Prevalence of timely initiation first ANC visit is known;*
- *Socio-demographic factors are associated with timely initiation of first ANC visit;*
- *Obstetric factors were independent predictors of timely initiation first ANC visit*.

### What this study adds

- *The prevalence of timely initiation first ANC was higher in the transformed districts than the non-transformed districts;*
- *Maternal knowledge and quality of maternal health service have positively reinforced timely initiation first ANC;*
- *District transformation has positive effect on timely initiation first ANC*.
